# Supplementary material for: A YAP-centered mechanotransduction loop drives collective breast cancer cell invasion
Source: Nat Commun. 2024 Jun 7;15:4866. doi: 10.1038/s41467-024-49230-z (PMC11161601; doi:10.1038/s41467-024-49230-z)
Supplement: Supplementary file 1 — Supplementary Information [file 41467_2024_49230_MOESM1_ESM.pdf]

A YAP-centered mechanotransduction loop drives collective breast cancer cell invasion

Antoine A. Khalil[1$], Daan Smits[1*], Peter D. Haughton[2*], Thijs Koorman[2*], Karin A. Jansen[2], Mathijs P. Verhagen[3], Mirjam van der Net[1], Kitty van Zwieten[1], Lotte Enserink[2], Lisa Jansen[1], Abdelrahman G. El-Gammal[1], Daan Visser[2], Milena Pasolli[1], Max Tak[1], Denise Westland[1], Paul J. van Diest[2], Cathy B. Moelans[2], M. Guy Roukens[1,4], Sandra Tavares[5], Anne-Marie Fortier[6], Morag Park[6], Riccardo Fodde[3], Martijn Gloerich[1], Fried. J.T. Zwartkruis[1], Patrick WB. Derksen[2$], Johan de Rooij[1$]

[1] Center for Molecular Medicine (CMM), University Medical Center Utrecht, The Netherlands

[2] Department Pathology, University Medical Center Utrecht, The Netherlands

[3] Department of Pathology, Erasmus Medical Center, Rotterdam, The Netherlands

[4] Regenerative Medicine Center Utrecht, University Medical Center Utrecht, Utrecht, The Netherlands

[5] i3S - Instituto de Investigação e Inovação em Saúde, Universidade do Porto, Porto, Portugal

[6] Goodman Cancer Institute McGill University, Depts Biochemistry and Oncology, McGill University, Goodman Cancer Institute, Montréal, Canada

* Equal contribution

$ Corresponding authors:

a.khalil@umcutrecht.nl, p.w.b.derksen@umcutrecht.nl, jderooij@y2y.eu

Supplementary Information

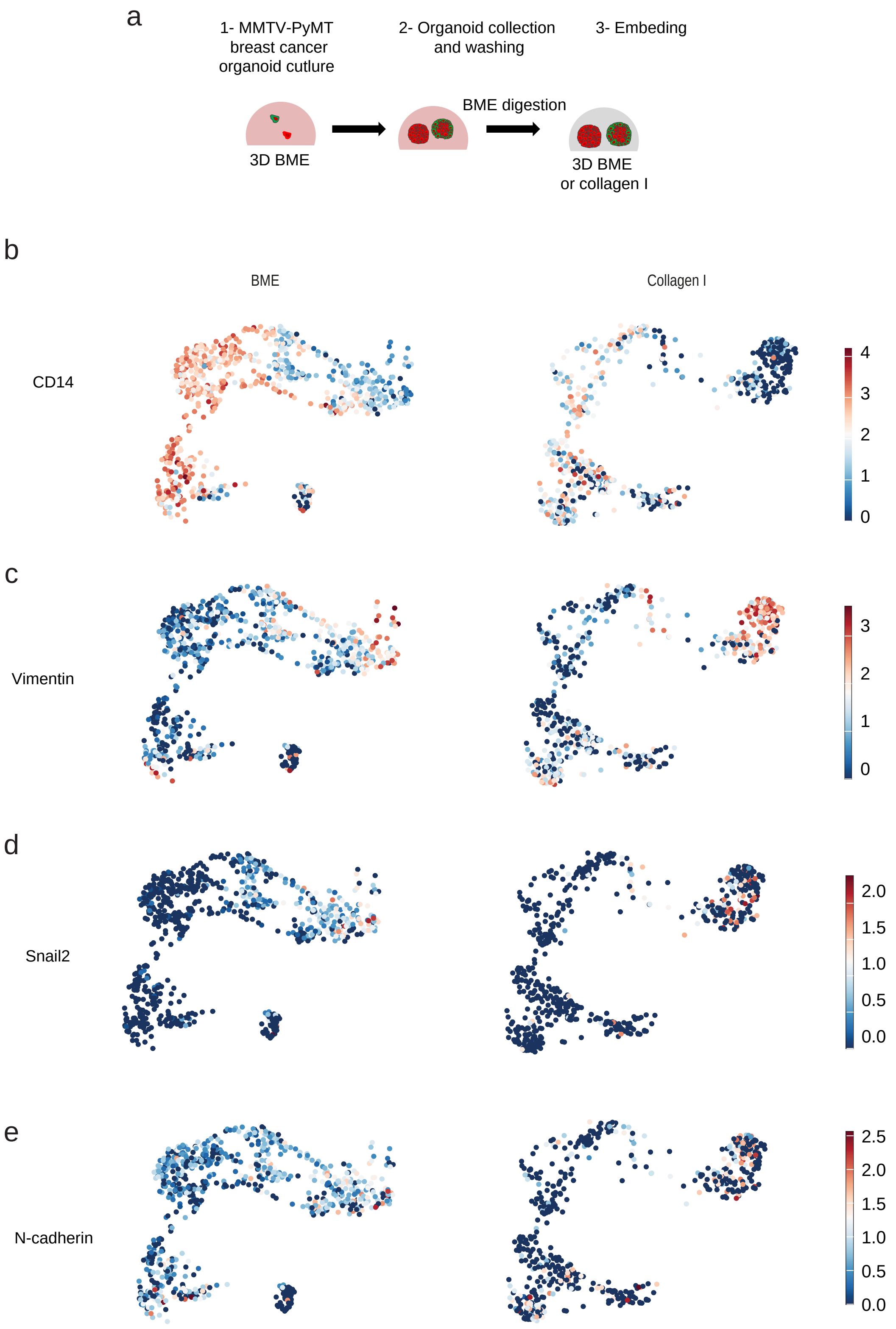

Supplementary Figure 1 (Related to Figure 2). (a) Strategy used to transfer organoids from 3D BME cultures to 3D Collagen I or BME for functional studies. (b-e) Feature plots from the single cell mRNA sequencing data from cells isolated from MMTV-PyMT organoids embedded in 3D BME or Collagen. Color scales indicate the normalized read counts of the luminal (c, Cd14) and mesenchymal markers (d-e, Vimentin, Snail2 and N-cadherin).

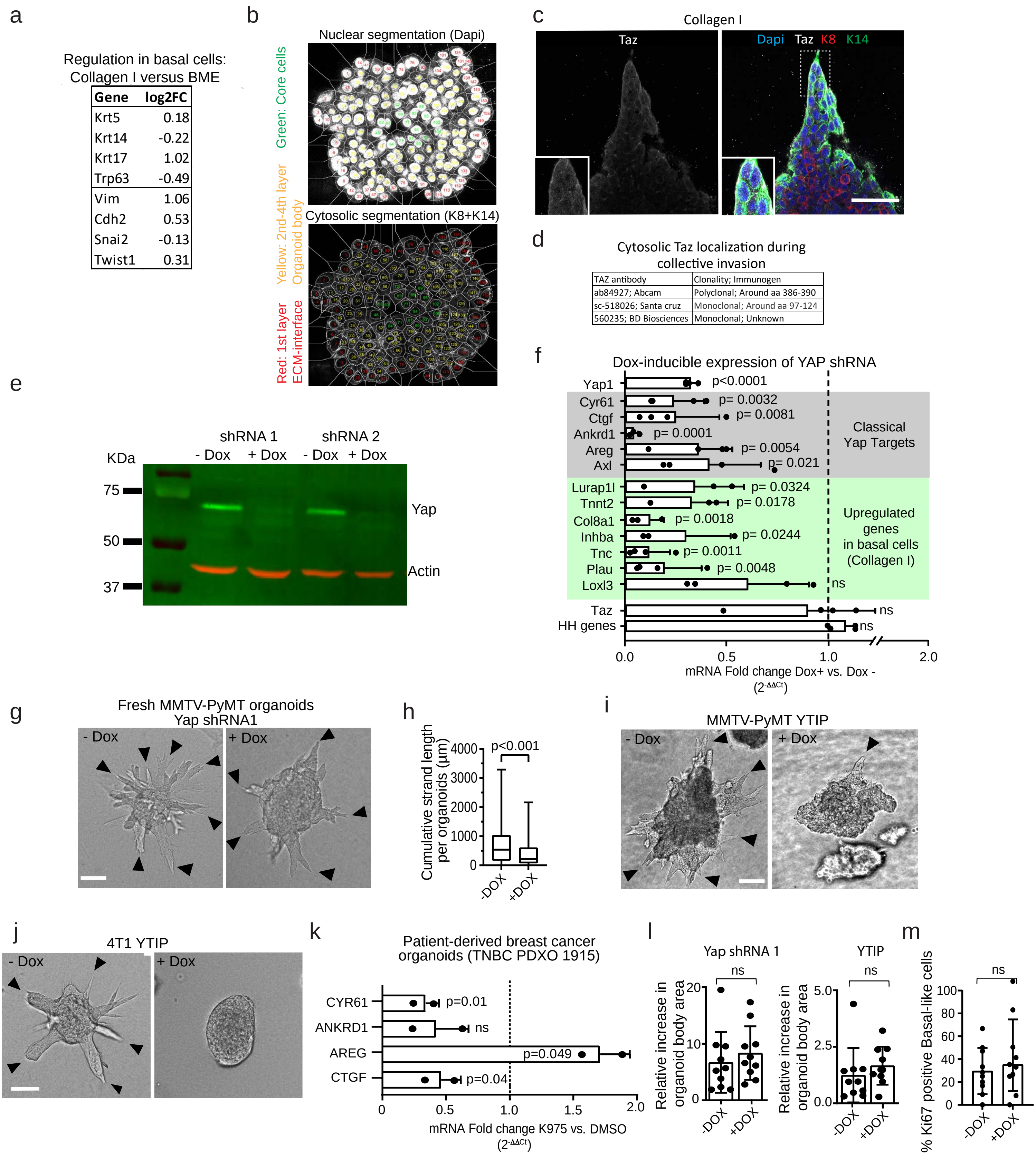
a
Regulation in basal cells: Collagen I versus BME
Gene log2FC
Krt5 0.18
Krt14 -0.22
Krt17 1.02
Trp63 -0.49
Vim 1.06
Cdh2 0.53
Snai2 -0.13
Twist1 0.31
b
Nuclear segmentation (Dapi)
Cytosolic segmentation (K8+K14)
Green: Core cells
Yellow: 2nd-4th layer Organoid body
Red: 1st layer ECM-interface
c
Collagen I
Taz
Dapi Taz K8 K14
d
Cytosolic Taz localization during collective invasion
TAZ antibody | Clonality; Immunogen
ab84927; Abcam | Polyclonal; Around aa 386-390
sc-518026; Santa cruz | Monoclonal; Around aa 97-124
560235; BD Biosciences | Monoclonal; Unknown
e
KDa
shRNA 1
shRNA 2
- Dox + Dox - Dox + Dox
75
50
37
Yap
Actin
f
Dox-inducible expression of YAP shRNA
Yap1 p<0.0001
Cyr61 p= 0.0032
Ctgf p= 0.0081
Ankrd1 p= 0.0001
Areg p= 0.0054
Axl p= 0.021
Classical Yap Targets
Lurap1l p= 0.0324
Tnnt2 p= 0.0178
Col8a1 p= 0.0018
Inhba p= 0.0244
Tnc p= 0.0011
Plau p= 0.0048
Loxl3 ns
Upregulated genes in basal cells (Collagen I)
Taz ns
HH genes ns
0.0 0.5 1.0 2.0
mRNA Fold change Dox+ vs. Dox - (2-ΔΔCt)
g
Fresh MMTV-PyMT organoids
Yap shRNA1
- Dox
+ Dox
h
p<0.001
Cumulative strand length per organoids (µm)
4000 3000 2000 1000 0
-DOX +DOX
i
MMTV-PyMT YTIP
- Dox
+ Dox
j
4T1 YTIP
- Dox
+ Dox
k
Patient-derived breast cancer organoids (TNBC PDXO 1915)
CYR61 p=0.01
ANKRD1 ns
AREG p=0.049
CTGF p=0.04
0 0.5 1.0 1.5 2.0
mRNA Fold change K975 vs. DMSO (2-ΔΔCt)
l
Yap shRNA 1
ns
Relative increase in organoid body area
20 10 0
-DOX +DOX
YTIP
ns
Relative increase in organoid body area
5.0 2.5 0.0
-DOX +DOX
m
ns
% Ki67 positive Basal-like cells
100 80 60 40 20 0
-DOX +DOX

Supplementary Figure 2 (Related to Figure 2 and 3). (a) List of basal and mesenchymal genes expressed in basal cells in Collagen I compared to basal cells in BME and corresponding Log2 fold change in mRNA expression. (b) Nuclear (based on dapi) and cytosolic (based on combination of K8+K14) image segmentation to analyze Yap nuclear/cytoplasmic ratios in K14 positive and negative cells within different locations in the organoids imaged by confocal microscopy. (c) Confocal imaging of Taz, K8, K14 in MMTV-PyMT organoids invading invading Collagen Representative image from 3 independent experiments. (c) Zoom in on invading strand and leader cell. (d) Summary of the TAZ specific antibodies used that show cytosolic distribution of TAZ in invasive strands of MMTV-PyMT organoids in Collagen I. (e) Western blot analysis of Yap expression and loading control (actin) from whole cell lysates derived from MMTV-PyMT organoid cultures after Yap knockdown using shRNA 1 and 2. Representative blot from 3 independent experiments. (f) qPCR data showing mRNA expression of the classical Yap targets and other genes (identified from single cell sequencing) in doxycycline treated (Dox+) normalized relative to control (Dox-) in MMTV-PyMT organoids harboring inducible Yap shRNA contructs, embedded in Collagen I for 3 days. Values represent average values with SD from at least three independent experiments. P values, Student's t-test. (g) Representative brightfield images of freshly isolated MMTV-PyMT organoids harboring dox-inducible Yap shRNA1 after 3 days in Collagen I in the absence and presence of dox. (h) Resulting cumulative length of the invasive strands in control and dox treated fresh MMTV-PyMT organoids, data represent the medians (black line), 25/75 percentiles (boxes) and maximum/minimum values (whiskers) from n=102 organoids from 3 independent experiments. (i, j) Representative brightfield images of MMTV-PyMT organoids (i) and 4T1 spheroids (j) harboring dox-inducible YTIP expression vector after 3 days in Collagen I in the absence and presence of dox. (k) qPCR data showing mRNA expression of the classical Yap targets in breast cancer patient-derived organoids embedded in Collagen I for 3 days, treated with K975 (5µM) normalized relative to control (DMSO). Values represent average values with SD from two independent experiments. (l) Effect of Yap knockdown or YTIP on growth of the organoid body. Values represent average cross section area relative to day 1 with SD from n=10 organoids per condition. (m) Ratio of basal-like cells scored positive for nuclear Ki67 in MMTV-PyMT organoids harboring dox-inducible YTIP expression vector after 3 days of culture in Collagen I. Average values with SD from n=10 organoids per condition from one experiment. (l, m) P values, two-tailed unpaired Mann-Whitney test. Scale bars: 50 µm (c, g, i, j), 10 µm (c, inset). Source data are provided as a Source Data file.

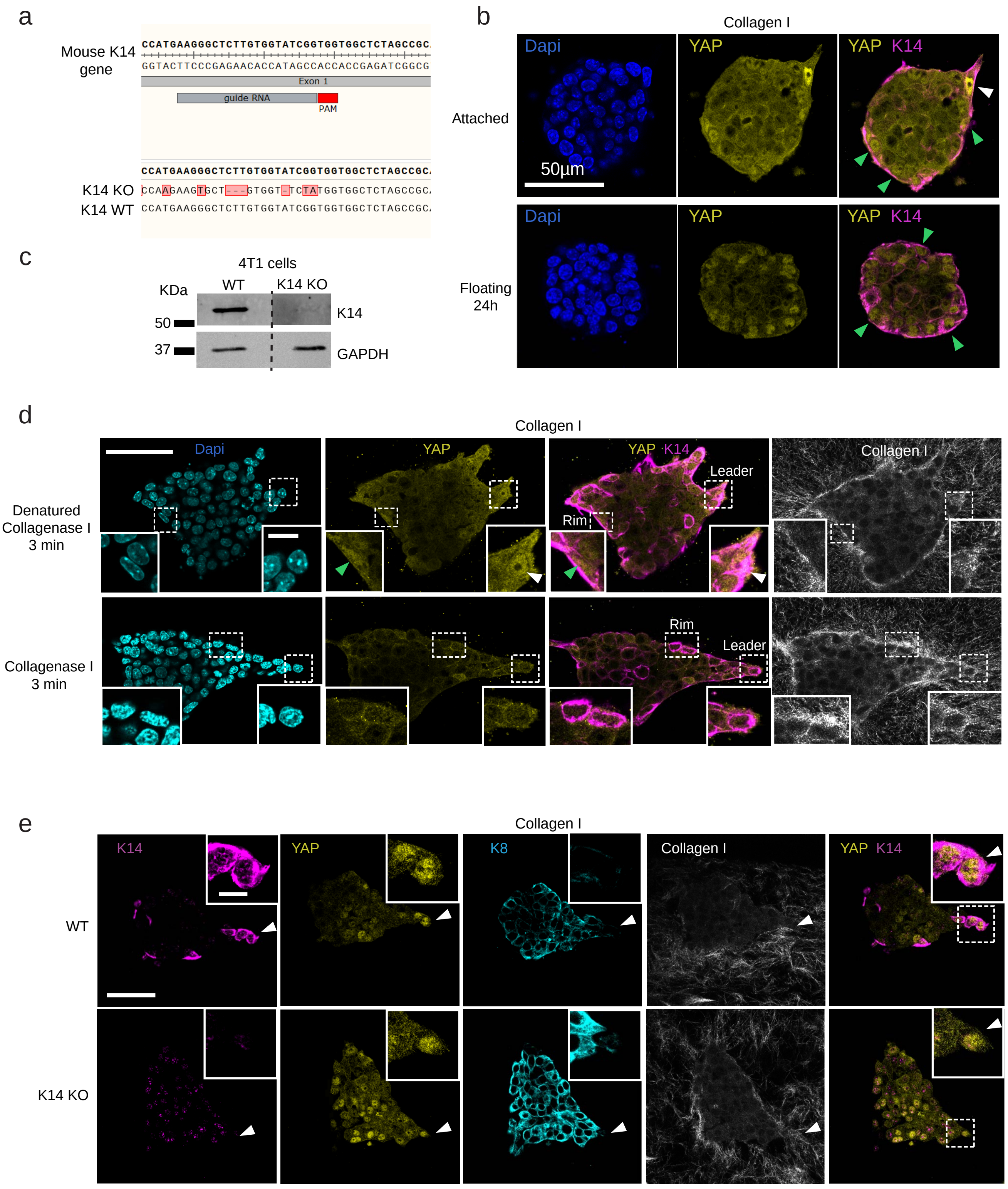

Supplementary Figure 3 (Related to Figure 4 and 5)**.** (a) Confirmation of K14 knockout (KO) in MMTV-PyMT organoids by Crispr-Cas9 gene editing. The guide RNA sequence targeting the 1st exon of the wild type (WT) mouse K14, dash (-) regions indicate the deleted nucleotides. (b) Yap distribution in rim and leader cells after 24 h in attached or floating Collagen gels. (c) Western blot analysis showing K14 and loading control (actin) from whole cell lysates derived from wild-type (WT) and K14 knockout (KO) 4T1 spheroid BME cultures. (d) Yap localization in basal rim cells and leader after partial degradation of the gel with collagenase. (e) Confocal imaging of K14, Yap and K8 in MMTV-PyMT WT and K14-KO organoids invading in Collagen I for 3 days. Arrowheads depict WT and K14-KO leader cells that maintain high nuclear signal Yap and low K8 signal. (b, d, e) Representative images from at least n=6 organoids from one experiment. Scale bars: 50 µm (b, d, e), 10 µm (inset: d, e). Source data are provided as a Source Data file.

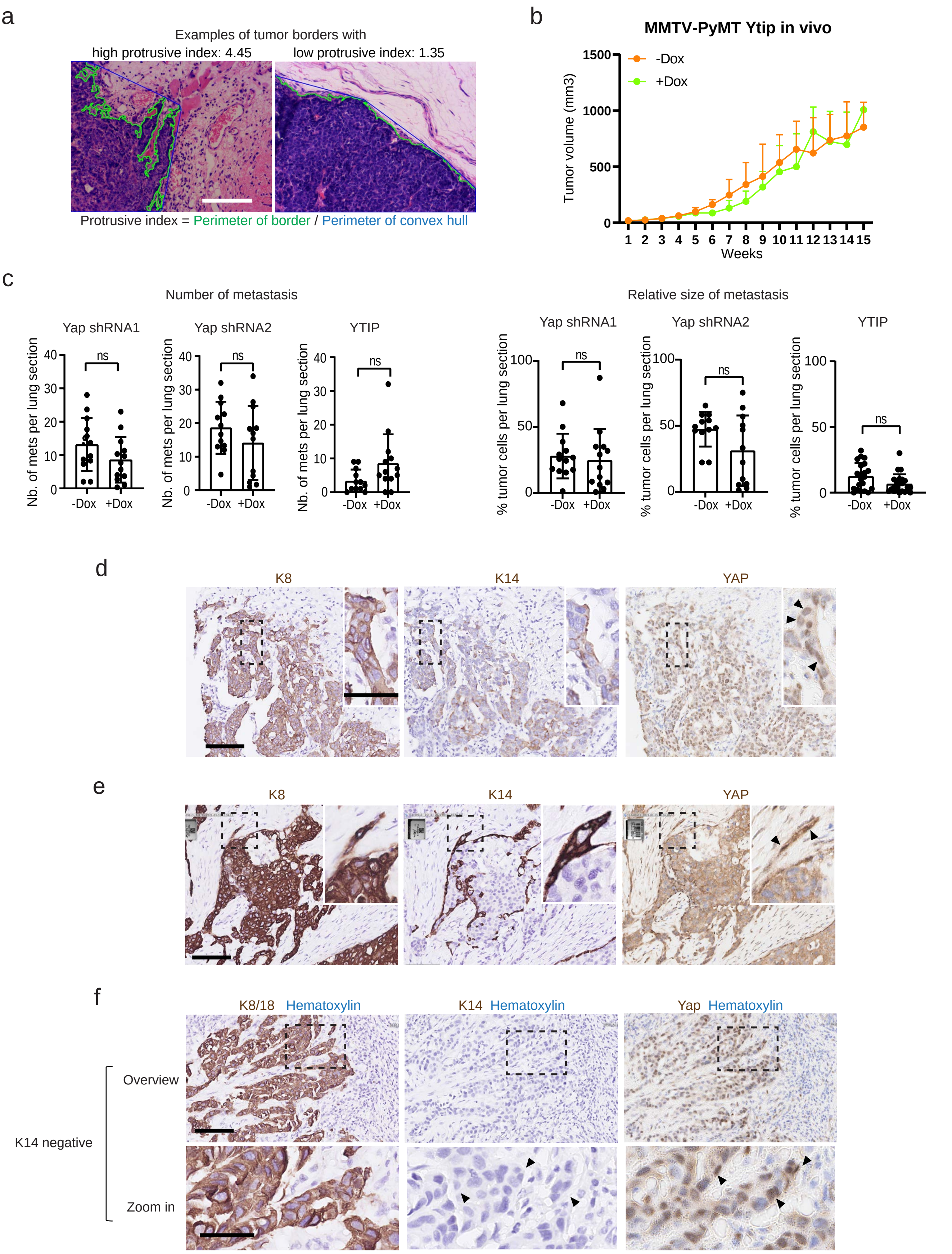

Supplementary figure 4 (Related to Figure 6)**.** (a) Example of the quantitative analysis of the morphology of the tumor borders and calculation of the protrusive index. (b) Tumor volumes (MMTV-PyMT YTIP) in supplemented with doxycycline (dox)-containing or control food. Values represent the average tumor volume with SD. (c) Number of metastasis per lung H&E section and relative tumor content per lung. Average values with SD from 12-13 mice per condition. P values, two-tailed unpaired Mann-Whitney test. (d-f) Representative images from IDC tissue sections that show K14 positive (n=4) (d, e) and negative (n=8) (f) invasive patterns. Black arrowheads depict positive signal for nuclear Yap. Scale bars: 50 µm (d-f inset), 100 µm (a, d-f). Source data are provided as a Source Data file.

Supplementary Table 1. Antibody list and information

Name	Species	Company	Dilutions	LOT#	Cat#	Species and relevant applications validated by the company	Species and relevant applications tested in published work	KO and KD Validation by our experiments using murine cells
YAP1, clone 63.7	Mouse	Santa Cruz	1:100 (IF)<br>1:1000 (WB)	E1619	sc-101199	Validated in murine and human cells by WB, IF, IHC	Murine cells: WB (Guo, et al., 2018, Cell Reports), Immunostaining (Elosegui-Artola, A., et al., 2017, Cell; Guo, L.., et al., 2018, JCB). IHC paraffin-embeded human tissue sections (Kaukonen, R., et al., 2016, Nature Communications; Liu, Y., et al., 2021, Cancer Science)	By YAP knockdown using multiple shRNA sequences by WB.
Keratin 8, TROMA-1	Rat	Developmental Studies Hybridoma Bank	1:50 (IF)	1/6/22 - 17μg/ml	AB_531826	Validated in murine and human cells by WB, IF, IHC	IF of murine cells (Bryja, V., et al., 2008, Cell Proliferation); IHC on paraffin embedded human tissue sections Wilson, M. R., Reske, J. J., et al., 2019, Nature Communications).	
Keratin 14 Polyclonal	Rabbit	BioLegend	1:400 (IF)<br>1:1000 (WB)	B374019	905301	Validated in human cells using IHC	IF of murine cells (Hannezo, E., Scheele, C. L. G. J., et al., 2017 Cell); IHC on paraffin embeded human tissue sections (I Larribere, L., et al, 2017 Stem Cell Reports).	By K14 KO using Crisrp/Cas9 by WB, IF
Cytokeratin 5, clone EP1601Y	Rabbit	Abcam	1:100 (IF)<br>1:1000 (WB)	GR157618-30	ab52635	Validated in murine and human cells by WB, IF, IHC		
Keratin 17, clone D73C7	Rabbit	Cell Signaling	1: 200 (IF)<br>1:500 (WB)	4543s	4543S	Validated in murine cells by WB, IF, IHC		By K17 KO using Crisrp/Cas9 by WB, IF
TAZ	Rabbit	Abcam	1:50 (IF)	GR257785-10	ab84927	Validated in murine and human cells by WB, IF, IHC	IF on hunan cells (Kandilya, D., et al., 2020, Scientific Report).	
Actin, clone C4	Mouse	Millipore	1:2500 (WB)	3845682	MAB1501	Validated in murine and human cells by WB, IF, IHC		
TAZ, clone D8	Mouse	Santa Cruz	1:25 (IF)	J2621	sc-518026	Validated in murine and human cells by WB, IF, IHC		
TAZ, clone M2-616	Mouse	BD Biosciences	1:100 (IF)	6138547	560235	Validated in murine and human cells by WB, IF, IHC		
P63, clone DAK-p63	Mouse	DAKO	1:50 (IF)	20079547	GA662	Validated in human cells using WB, IF, IHC		
GAPDH	Mouse	EMD Millipore	1:5000 (WB) (WB)	3725986	MAB374	Validated in murine and human cells using WB		

IF: Immunofluorescence, WB: Western Blot, IHC: Immunohistochemistry

Supplementary Table 2. Mouse and human primers used for qPCR

**Gene (mouse)**	**Sequence Fwd primer (5' à 3')**	**Sequence Rev primer (5' à 3')**
Yap1	CCTCGTTTTGCCATGAACCAG	GCTGCTGCTGGTTGGAACTG
Ctgf	GGAGAACTGTGTACGGAGCGT	CCATCTTTGGCAGTGCACAC
Ankrd1	GCTGGTAACAGGCAAAAAGAAC	CCTCTCGCAGTTTCTCGCT
Cyr61	GAAAGGGATCTGCAGAGCTCAG	GCACTGGTGTTTACAGTTGG
Areg	GGTGGTGACATGCAATTGTC	GCAATCTTGGATAGGTCCTTG
Krt14	AGCGGCAAGAGTGAGATTTCT	CCTCCAGGTTATTCTCCAGGG
Vgll4	TGTGAAAACGACCACGTCTCC	GCAGTCTCCGTTGACAGTCTTAT
Plau	GCGCCTTGGTGGTGAAAAAC	TTGTAGGACACGCATACACCT
Hbegf	CGGGGAGTGCAGATACCTG	TTCTCCACTGGTAGAGTCAGC
Wwtr1	CACCCGTCCATCACTTCCAC	TTCATTGCGAGATTCGGCTG
Axl	CCAGGAACTGCATGCTGAAT	GGCGGTAGTAATCCCCGTTG
Itga2	AGGGTACCATTCGCACCAAG	CACCATAGCCATCCAGGGAC
Inhba	TCCGAAGGATGGACCTAACTC	GCTTTCTGATCGCGTTGAGAAG
Tnc	TTTGCCCTCACTCCCGAAG	AGGGTCATGTTTAGCCCACTC
Pbgd	GCCTACCATACTACCTCCTGGCT	AAGACAACAGCATCACAAGGGTT
Hnrnpa	TGACAGCTATAACAACGGAG	AAAGTTTCCTCCCTTCATCG
Tuba1	AGGATTATGAGGAGGTTGGT	ATAAACATCCCTGTGGAAGC
Cyca	ACTGAATGGCTGGATGGCAA	CAAAACGCTCCATGGCTTCC
**Gene (human)**	**Sequence Fwd primer (5' à 3')**	**Sequence Rev primer (5' à 3')**
CYR61	GATCTGCAGAGCTCAGTCAGAG	CCATCAATACATGTGCACTG
YAP1	TCCTTAACAGTGGCACCTATC	GGAAACGGTTCTGCTGTGAG
CTGF	CTGGAAGAGAACATTAAGAAGG	GGTATGTCTTCATGCTGGTG
AREG	GAGCCGACTATGACTACTCAGA	TCACTTTCCGTCTTGTTTTGGG
ANKRD1	AGTAGAGGAACTGGTCACTGG	TGTTTCTCGCTTTTCCACTGTT
GAPDH	CATTTCCTGGTATGACAACG	CTCTTCCTCTTGTGCTCTTG

Supplementary Figure 2e

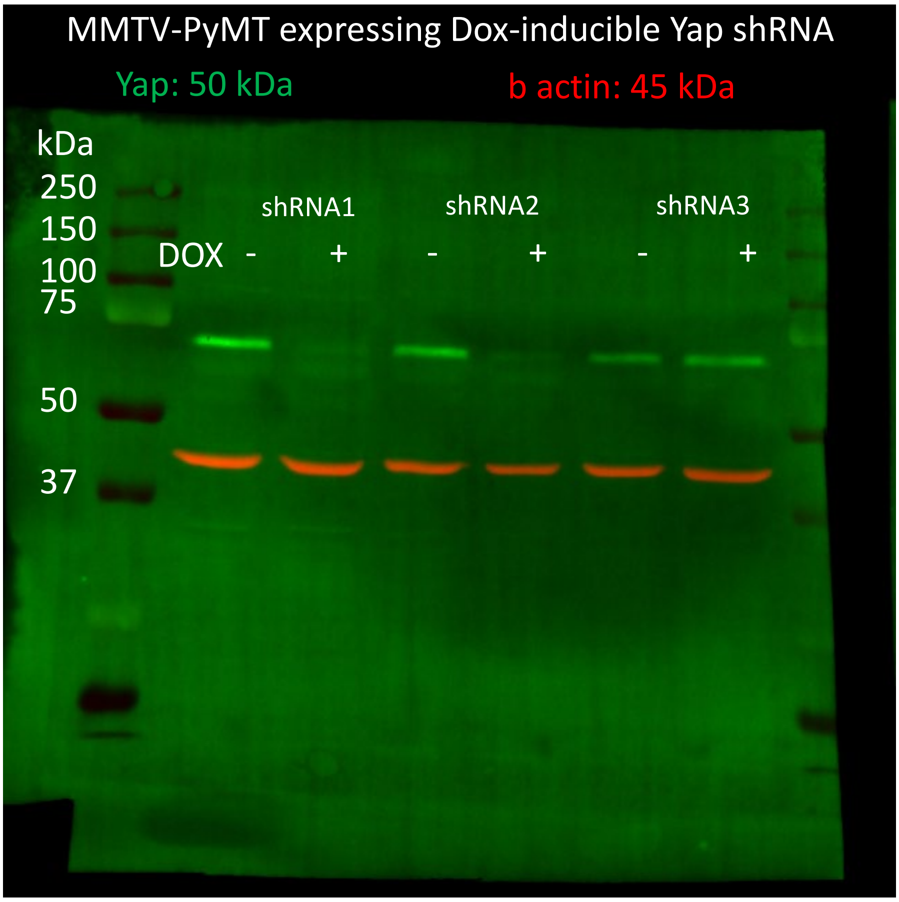

Supplementary Figure 3c

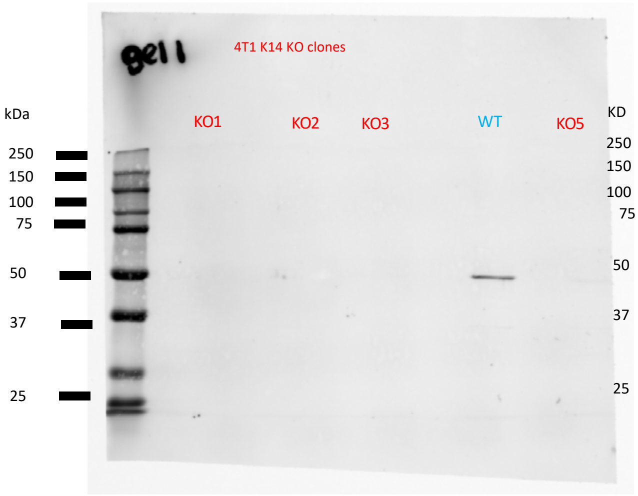

Keratin 14: 50 kDa

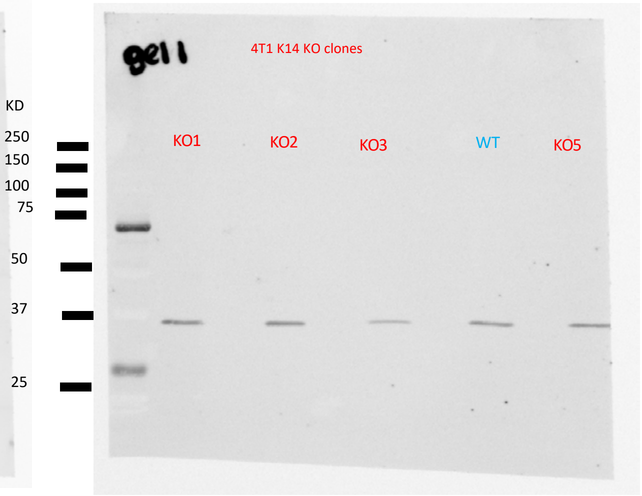

GAPDH: 36 kDa
