# Supplementary material for: A YAP-centered mechanotransduction loop drives collective breast cancer cell invasion
Source: Nat Commun. 2024 Jun 7;15:4866. doi: 10.1038/s41467-024-49230-z (PMC11161601; doi:10.1038/s41467-024-49230-z)
Supplement: Supplementary file 3 — Description of Additional Supplementary Files [file 41467_2024_49230_MOESM3_ESM.pdf]

Description of Additional Supplementary Files

Supplementary Data 1 - Single cell mRNA sequencing data displaying all the genes regulated in basal-like cells in collagen I and BME conditions

Supplementary Movie 1. Time lapse confocal imaging of MMTV-PyMT organoids expressing endogenously tagged K14-GFP during leader cell formation and collective invasion in 3D Collagen I.

Field size: 465.58 μm x 232.79 μm. The playback speed is indicated in the time stamp of the video.

Supplementary Movie 2. Time lapse confocal imaging of MMTV-PyMT organoids expressing Histone 2b mNeon during leader cell formation and collective invasion in 3D Collagen I. Field size: 354.25 μm x 354.25 μm. The playback speed is indicated in the time stamp of the video.

Supplementary Movie 3. Time lapse confocal imaging of fluorescent bead displacement by mixed

MMTV-PyMT organoids harboring YAP shRNA +/-DOX in 3D Collagen I. Field size: 820 μm x 362 μm.The playback speed is indicated in the time stamp of the video.

Supplementary Movie 4. Time lapse confocal imaging of fluorescent bead displacement by luminal MMTV-PyMT organoids harboring YAP 5SA +/-DOX in 3D Collagen I. Field size: 1214.56 μm x 607.29 μm. The playback speed is indicated in the time stamp of the video.
