# Supplementary material for: A YAP-centered mechanotransduction loop drives collective breast cancer cell invasion
Source: Nat Commun. 2024 Jun 7;15:4866. doi: 10.1038/s41467-024-49230-z (PMC11161601; doi:10.1038/s41467-024-49230-z)
Supplement: Supplementary file 9 — Reporting Summary [file 41467_2024_49230_MOESM9_ESM.pdf]

nature portfolio

Corresponding author(s): Antoine Khalil, Patrick Derksen, Johan de Rooij

Last updated by author(s): May 15, 2024

Reporting Summary

Nature Portfolio wishes to improve the reproducibility of the work that we publish. This form provides structure for consistency and transparency in reporting. For further information on Nature Portfolio policies, see our Editorial Policies and the Editorial Policy Checklist.

Statistics

For all statistical analyses, confirm that the following items are present in the figure legend, table legend, main text, or Methods section.

n/a	Confirmed	
☐	☒	The exact sample size (*n*) for each experimental group/condition, given as a discrete number and unit of measurement
☐	☒	A statement on whether measurements were taken from distinct samples or whether the same sample was measured repeatedly
☐	☒	The statistical test(s) used AND whether they are one- or two-sided<br>*Only common tests should be described solely by name; describe more complex techniques in the Methods section.*
☒	☐	A description of all covariates tested
☐	☒	A description of any assumptions or corrections, such as tests of normality and adjustment for multiple comparisons
☐	☒	A full description of the statistical parameters including central tendency (e.g. means) or other basic estimates (e.g. regression coefficient) AND variation (e.g. standard deviation) or associated estimates of uncertainty (e.g. confidence intervals)
☐	☒	For null hypothesis testing, the test statistic (e.g. *F*, *t*, *r*) with confidence intervals, effect sizes, degrees of freedom and *P* value noted<br>*Give P values as exact values whenever suitable.*
☒	☐	For Bayesian analysis, information on the choice of priors and Markov chain Monte Carlo settings
☒	☐	For hierarchical and complex designs, identification of the appropriate level for tests and full reporting of outcomes
☒	☐	Estimates of effect sizes (e.g. Cohen's *d*, Pearson's *r*), indicating how they were calculated

Our web collection on statistics for biologists contains articles on many of the points above.

Software and code

Policy information about availability of computer code

Data collection	Zen black (2.3 sp1), EVOS M5000, Nanozoomer Digital Pathology Software (NDP 2.6.17).
Data analysis	Graph Pad Prism 8, MATLAB R2018a, Fiji (ImageJ 1.53f51), Python (perimeter correction), HOMER (homer.ucsd.edu)

For manuscripts utilizing custom algorithms or software that are central to the research but not yet described in published literature, software must be made available to editors and reviewers. We strongly encourage code deposition in a community repository (e.g. GitHub). See the Nature Portfolio guidelines for submitting code & software for further information.

Data

Policy information about availability of data

All manuscripts must include a data availability statement. This statement should provide the following information, where applicable:

- Accession codes, unique identifiers, or web links for publicly available datasets
- A description of any restrictions on data availability
- For clinical datasets or third party data, please ensure that the statement adheres to our policy

Single cell sequencing data were deposited into the Gene Expression Omnibus database under accession number GSE197821 and are available at the following URL: https://www.ncbi.nlm.nih.gov/geo/query/acc.cgi?acc=GSE197821.
Due to the sizes of the files, the raw data from confocal microscopy, immunohistochemical analysis and qPCR were not provided and will be given upon request from the corresponding author.

A complete data availability statement is provided in the manuscript under 'data availability' section.

Research involving human participants, their data, or biological material

Policy information about studies with human participants or human data. See also policy information about sex, gender (identity/presentation), and sexual orientation and race, ethnicity and racism.

Reporting on sex and gender	Tissue sections from primary tumors were taken from female patients with breast cancer (invasive ductal carinoma). The Patient-derived cancer organoid (PDXO 1915) was established from a primary breast tumor isolated from a female patient.
Reporting on race, ethnicity, or other socially relevant groupings	No socially constructed or socially relevant categorization variables were used
Population characteristics	Breast cancer tissue sections diagnosed as high grade invasive ductal carcinoma (IDC) with different molecular subtypes (Luminal A, Luminal B, and Triple Negative). The age of the patients ranged from 30-75 years.
Recruitment	Patients with high grade tumors were selected, which has no potential self-selection biases.
Ethics oversight	The use of patient material was approved by the Tissue Science Committee of the University Medical Center Utrecht and informed consent was obtained from the participants. Banking of human specimens and associated clinical data is approved by MUHC research ethics board (study approval SUR-2000-966 and SUR-99-780). All patient data and biological samples were obtained from patients at the MUHC after obtaining informed consent.

Note that full information on the approval of the study protocol must also be provided in the manuscript.

Field-specific reporting

Please select the one below that is the best fit for your research. If you are not sure, read the appropriate sections before making your selection.

☒ Life sciences ☐ Behavioural & social sciences ☐ Ecological, evolutionary & environmental sciences

For a reference copy of the document with all sections, see nature.com/documents/nr-reporting-summary-flat.pdf

Life sciences study design

All studies must disclose on these points even when the disclosure is negative.

Sample size	For in vitro studies sample size was determined empirically based on our previous published studies in similar experimental contexts (Khalil AA et al., 2020; Koorman at al., 2023). For in vivo studies the number of mice was determined using power analyses based on one-way ANOVA (a-error of 5% and a power of 0.9; G~power software).<br>Except for the qPCR and Collagen alignment analysis, statistical significance between groups in all experiments was determined using the two-tailed unpaired Mann–Whitney (two groups) or Kruskal–Wallis test (more than two groups) with Dunns' correction. For the qPCR and collagen alignment data student t-test was performed after confirming normal distribution of the data by D'Agostino and Pearson omnibus normality test.
Data exclusions	Occasional organoids showing cytotoxic features (cellular debris, condensed nuclei) and organoids growing on the 2D bottom of the culture plate were excluded from further analysis. Tissue sections from in vivo experiments that are detached from the slides, show no tumor, is highly necrotic tumor were excluded.
Replication	The number of replicates is stated in the figure legends. The graphs of almost all experiments originate from at least three replicas, except for qPCR from the PDXO 1915 patient-derived organoids which were derived from 2 independent experiments and the graphs showing the effect of K14 KO on invasion and effect of Yap KD/OE on collagen I alignment and bead displacement, which are derived from 1 experiment each.
Randomization	Organoids (in vitro experiments) and mice (in vivo experiments) were randomly allocated to the different experimental groups.
Blinding	Functional experiments in vitro were not blinded, partly because image analysis was performed using unbiased criteria and partly because the doxycycline-treated organoids (Yap shRNA1 and YTIP) were distinguishable from the control groups. For in vivo experiments the identity of organoids (harboring Yap shRNA1 or Yap shRNA2 or YTIP) injected into the mammary fat pad as well as image acquisition of tumor borders were blinded. For image analysis, blinding was not necessary since the images were analyzed using an automated unbiased approach.

Reporting for specific materials, systems and methods

We require information from authors about some types of materials, experimental systems and methods used in many studies. Here, indicate whether each material, system or method listed is relevant to your study. If you are not sure if a list item applies to your research, read the appropriate section before selecting a response.

Materials & experimental systems

n/a	Involved in the study
☐	☒ Antibodies
☐	☒ Eukaryotic cell lines
☒	☐ Palaeontology and archaeology
☐	☒ Animals and other organisms
☒	☐ Clinical data
☒	☐ Dual use research of concern
☒	☐ Plants

Methods

n/a	Involved in the study
☒	☐ ChIP-seq
☒	☐ Flow cytometry
☒	☐ MRI-based neuroimaging

Antibodies

Antibodies used: All antibodies used in this study, their dilution and validation are listed in Supplementary Table 1.

Validation: All antibodies used in this study, their dilution and validation are listed in Supplementary Table 1.

Eukaryotic cell lines

Policy information about cell lines and Sex and Gender in Research

Cell line source(s):
- MMTV-PyMT organoids expressing endogenously tagged E-cadherin CFP provided by Prof. Jacco van Rheenen (NKI, Amsterdam) (Beerling E. et al., 2016).
- The 4T1 metastatic breast cancer cell line (CRL-2539 ATCC).
- Patient-derived breast cancer organoids PDXO 1015-2 provided by Prof. Morag Park (Mc Gill University, Canada) (Savage P et al., 2020).

Authentication:
- MMTV-PyMT were authenticated by confirming the expression of E cadherin-CFP by fluorescent microscopy and western blot analysis.
- The identity of 4T1 cells and PDXO 1915 patient-derived organoids were verified by short tandem repeat DNA profiling.

Mycoplasma contamination: Organoid and cell lines were tested negative for mycoplasma (MycoAlert Mycoplasma Detection Kit, Lonza).

Commonly misidentified lines (See ICLAC register): No commonly misidentified cell lines were used in this study.

Animals and other research organisms

Policy information about studies involving animals; ARRIVE guidelines recommended for reporting animal research, and Sex and Gender in Research

Laboratory animals: Athymic Nude- Foxn1nu, Female mice, Age 6-9 weeks, Temperature room: between 20-24?; Huminidty room Between 45-65%; Dark/ light cycle: From 7pm-7am lights off, from 7am-7pm lights on.

Wild animals: No wild animals were used in this study.

Reporting on sex: Female mice were used with mammary glands, to model breast cancer in vivo.

Field-collected samples: The study did not involve samples collected from the field.

Ethics oversight: All animal experiments were performed in accordance with local, National and European guidelines under permit AVD1150020209964 issued by The Netherlands Food and Consumer Product Safety Authority (NVWA) of the ministry of Agriculture, Nature and Food.

Note that full information on the approval of the study protocol must also be provided in the manuscript.
